# Comparing Dietary Intake and Cardiovascular Risk Factors in Vancouver’s South Asian Community

**DOI:** 10.3390/nu17121967

**Published:** 2025-06-10

**Authors:** Rehan Jessa, Rachel A. Murphy, Nadia A. Khan, Tricia S. Tang

**Affiliations:** 1 Faculty of Medicine, The University of British Columbia, Vancouver, BC V6T 1Z3, Canada; rjessa@student.ubc.ca; 2 School of Population and Public Health, Department of Medicine, The University of British Columbia, Vancouver, BC V6T 1Z3, Canada; rachel.murphy@ubc.ca; 3 Division of General Internal Medicine, Department of Medicine, The University of British Columbia, Vancouver, BC V6Z 1Y6, Canada; nakhanubc@gmail.com; 4 Division of Endocrinology, Department of Medicine, The University of British Columbia, Vancouver, BC V5Z 1M9, Canada

**Keywords:** South Asian, dietary intake, vegetarian, omnivores

## Abstract

**Background:** Compared to omnivorous diets, vegetarian diets are generally linked to improved body weight, blood pressure, lipid profiles, and glycemic control. Despite having the highest global prevalence of vegetarianism, South Asians in Canada exhibit disproportionately high rates of cardiovascular disease (CVD) and diabetes. This study examines the usual dietary intake and CVD risk factors among South Asian vegetarians and omnivores at risk of diabetes in British Columbia, Canada. **Methods:** Of a community sample of 100 South Asian adults at high risk of diabetes and recruited from 12 faith-based centers across the Metro Vancouver area, 96 completed the culturally adapted 163-item SHARE Food Frequency Questionnaire to determine their dietary status. CVD risk factors such as body mass index (BMI) and waist circumference (WC) were also assessed. The usual dietary intake metrics, including the total energy, macronutrient, and micronutrient consumption, were compared between vegetarians and omnivores. The associations between diet type, BMI, and WC were analyzed. **Results:** Of the 96 participants, 50 identified as vegetarians and 46 as omnivores. The mean age was similar between groups: 64.9 (±9.0) years for vegetarians and 65.6 (±10.5) years for omnivores. Females comprised a higher proportion of vegetarians (54.0% vs. 34.8%). Vegetarians reported a greater intake of carbohydrates and foods with a higher glycemic index and glycemic load. The fat intake was comparable between groups. Omnivores had a significantly higher intake of niacin, vitamin B-12, potassium, and zinc. Both groups exceeded the recommended sodium intake. Overall, 90.6% of the participants were classified as overweight or obese, with no significant association between vegetarianism and reduced adiposity. **Conclusions:** Both dietary groups exhibited an increased prevalence of overweight and obesity and had nutritional profiles that may be associated with elevated cardiometabolic risk. Factors such as dietary acculturation and a suboptimal diet quality may underlie these findings. Culturally tailored nutritional interventions are warranted to address the specific needs of South Asian Canadian communities.

## 1. Introduction

As of 2024, it has been estimated that up to 30% of the Canadian population is living with diabetes [1]. South Asians (SAs) are the largest and fastest-growing immigrant group in Canada and represent up to 16% of diabetes cases nationwide [1]. Unfortunately, SA adults are at greater risk of developing diabetes and cardiovascular disease (CVD) compared to that in other ethnic groups [2,3,4,5,6,7]. Compared to White Caucasians of a similar body weight and body mass index (BMI), SAs are more likely to experience greater abdominal adiposity, insulin resistance, hyperglycemia, and dyslipidemia, each of which is a significant risk factor for diabetes and CVD [2,3,4,5,6,7,8,9].

Diet is a modifiable lifestyle factor with a role in preventing and managing cardiometabolic risk factors [10,11,12,13]. Specifically, total energy intake and the dietary composition of macronutrients and micronutrients, as well as the quantity of processed foods and fiber, are important dietary factors that can impact blood glucose levels, metabolism, and consequentially the development of cardiometabolic risk factors. For this reason, vegetarian and plant-based diets have been promoted and emphasized due to their traditionally healthier dietary composition and their consistent associations with a lower risk of all-cause mortality compared to that under standard diets. Historically, adherence to vegetarianism has been shown to significantly reduce CVD risk across countries and ethnic groups [14,15,16]. Compared to individuals who consume animal products, those adhering to a vegetarian diet tend to have a lower body weight, a reduced blood pressure, improved lipid profiles, and better glycemic control [16,17,18,19]. As such, it is reasonable to consider that vegetarianism could be an effective strategy for lowering diabetes risk and preventing cardiovascular complications.

Vegetarianism is widely observed among many South Asians due to cultural, religious, and traditional beliefs [20]. However, with increasing Westernization, the traditional South Asian vegetarian diet, once rich in vegetables, whole grains, legumes, and lentils, has shifted toward less healthy patterns [21,22,23]. As a result, contemporary South Asian vegetarian diets increasingly feature a higher carbohydrate intake and the greater consumption of unhealthy refined and processed foods rich in fats, oils, salt, and sugars [21,22,23,24]. Not surprisingly, the health benefits of vegetarian diets among South Asians have been inconsistent [21,22,23,24,25]. For example, SA vegetarians in the United States (US) have been found to have lower BMIs [22,23], waist circumferences (WCs) [23], and blood lipid levels [22,23,25] and improved glycemic control [21,23] compared to those in omnivores but have also been reported to have a similar risk of CVD and metabolic syndrome compared to that in individuals who consume animal products [21,26]. Despite following a vegetarian diet, South Asians exhibit a greater risk of CVD than that in vegetarians of other ethnicities, which may be attributable to specific dietary practices [27].

Variations in the dietary intake among South Asian populations at the national and global levels may significantly affect the incidence and outcomes of both acute and chronic diseases [21,22,23,24,28]. There remains a lack of comprehensive research on the dietary composition and health implications of vegetarian diets among South Asians residing in Canada.

To expand the limited evidence base in this population and assess the potential role of a vegetarian diet in reducing diabetes risk, this study investigates and compares the usual dietary intake (total energy intake, macronutrients, and micronutrients) and CVD risk factors in SA adult vegetarians and omnivores living in Vancouver, British Columbia (BC), Canada.

## 2. Materials and Methods

Approved by the University of British Columbia (H13-00189) Clinical Research Ethics Board and the Fraser Health Research Ethics Board (FHREB 2013–030), this study is part of a larger investigation (Prevention Matters) examining the CVD risk factors and lifestyle behaviors in SA adults at risk of diabetes. A more comprehensive description of the study’s methodology has previously been reported [29]. Briefly, a cross-sectional convenience sample of SA adults was recruited from faith-based centers (Gurdwaras and Mandirs) in the Metro Vancouver area between July 2013 and June 2014. Participants were eligible for inclusion in this study if they met the following criteria: they (1) self-identified as SA, (2) were ≥21 years of age, (3) had no previous diagnosis of diabetes, (4) spoke Punjabi and/or English, (5) lived in the Metro Vancouver area, and (6) were noted to be at increased risk of diabetes (a score ≥ 5 out of 11 points) based on the 7-item American Diabetes Association (ADA) diabetes risk test [30]. Of the 425 SA adults from the larger study, a sample of 100 participants was selected for comprehensive dietary assessment in the present study, and 96 were included in the final data analysis. Participants completed a self-reported survey that assessed their sociodemographic backgrounds and usual dietary intake. Height, weight, and waist circumference measurements were taken, and their body mass indexes were calculated.

### 2.1. Measures

Sociodemographic information was collected through a 14-item questionnaire, including data on age, sex, country of birth, parental (mother’s and father’s) country of birth, number of years lived in Canada (for immigrants), annual household income, highest educational attainment, languages spoken, religious affiliation, marital status, and employment status.

Dietary intake was measured using the culturally tailored Study of Health and Risk in Ethnic groups Food Frequency Questionnaire (SHARE FFQ), a 163-item dietary assessment tool that includes 61 food items selected and validated by South Asian community members in Canada with ancestral origins in India, Pakistan, Sri Lanka, and Bangladesh [31]. Participants were asked to report the frequency of consumption and portion size for each food item consumed in a typical day over the previous 12 months. The SHARE FFQ also classified dietary status using predefined vegetarian categories. Participants were categorized as vegetarians if they reported following a vegan diet (excluding dairy, eggs, meat, poultry, and seafood), a lacto-vegetarian diet (including dairy but excluding eggs, meat, poultry, and seafood), or a lacto-ovo-vegetarian diet (including both dairy and eggs but excluding meat, poultry, and seafood). Conversely, participants were classified as omnivores if they identified as semi-vegetarian (occasionally consuming meat, poultry, or seafood), as consuming chicken and fish but not red meat, or as non-vegetarian (consuming dairy, eggs, meat, poultry, and seafood).

The CVD risk factors assessed in this study included BMI and WC. Height was measured using a portable stadiometer (Seca 213), and weight was measured using a portable electronic scale (Seca 874). WC was measured using a flexible Seca measuring tape positioned at the midpoints between the lower rib margin and the iliac crest. BMI was calculated using measured height and weight (kg/m^2^). Measures of physical activity [32] and psychosocial factors [29] were evaluated in the broader Prevention Matters cohort but were not included in the present analysis.

### 2.2. Statistical Methods and Data Analysis

Participants were categorized as either vegetarian or omnivorous based on their responses to the vegetarian status question included in the SHARE FFQ. Continuous sociodemographic variables and CVD risk factors (BMI and WC) were compared between the vegetarians and omnivores using the two-sample t-test and Wilcoxon’s Rank Sum test, while categorical sociodemographic variables were analyzed using the Chi-square test and Fisher’s exact test. For the continuous sociodemographic variables, the results were reported as the mean ± standard deviation (SD), with additional reporting of the median and interquartile range (IQR) for age, BMI, and WC. Frequencies and percentages were used to describe categorical variables.

To assess the differences in the daily macronutrient and micronutrient intake between the vegetarians and omnivores, linear regression models adjusted for age, sex, and total caloric intake were used. For each nutrient, the estimated mean intake, 95% confidence intervals, and *p*-values were calculated. A sensitivity analysis was also conducted using log-transformed macronutrient and micronutrient values to normalize the distributions and address outliers. Outliers were defined as observations falling below the 25th percentile or above the 75th percentile plus two times the IQR. Differences in the proportion of total energy obtained from macronutrients (protein, carbohydrates, and fat) were evaluated using the two-sample *t*-test.

Due to sample size limitations, participants classified as overweight and obese were analyzed together as a single overweight/obese group. The weight categories were defined using two sets of criteria: first, the India Consensus Statement for Diagnosis of Obesity, Abdominal Obesity and the Metabolic Syndrome (ICS-DOAMS), which defines a normal BMI as 18.0–22.9 kg/m^2^, overweight as 23.0–24.9 kg/m^2^, and obesity as >25.0 kg/m^2^, with abdominal obesity defined as a WC ≥ 90 cm for males and ≥80 cm for females [33], and second, the World Health Organization (WHO) BMI classifications were applied [34]. To explore the associations between diet and BMI, logistic regression models were constructed and adjusted for age and sex. Odds ratios (ORs) and 95% confidence intervals were reported to estimate the likelihood of following a vegetarian diet relative to an omnivorous diet and being overweight/obese relative to a normal weight. The omnivorous diet group served as the reference category. All statistical analysis were performed using SAS version 9.4 statistical software.

## 3. Results

### 3.1. Sociodemographic Characteristics

The sociodemographic characteristics of the 96 participants (of 100) who completed the survey and the full SHARE FFQ are shown in Table 1. Four participants were excluded, as they did not complete the full SHARE FFQ. Of the 96 participants, 50 were vegetarian and 46 were omnivores. There was no significant difference between vegetarians and omnivores in their mean age; mean years living in Canada; sex; education; or income.

### 3.2. Usual Dietary Intake

Overall, vegetarians appeared to consume more carbohydrates and less protein, less dietary cholesterol, and fewer of several micronutrients compared to omnivores. Table 2 presents the age- and sex-adjusted mean total energy intake, as well as the macronutrient and micronutrient intakes further adjusted for age, sex, and the mean total energy intake using linear regression models. Table 3 provides the results from the sensitivity analysis, including the mean total energy intake, macronutrient intake, and micronutrient intake (i.e., linear regression with log transformation excluding outliers).

### 3.3. Caloric Intake

There was no statistically significant difference in the mean total energy intake between vegetarians and omnivores [1937.8 kcal (95% CI: 1793.5, 2082.2) vs. 2068.98 kcal (95% CI: 1915.4, 2222.60); *p* = 0.22]. This finding remained non-significant in the sensitivity analysis (*p* = 0.23).

### 3.4. Macronutrient Intake

#### 3.4.1. Protein

Table 4 displays the estimated proportions of the daily caloric intake derived from protein, carbohydrates, and fats for both vegetarians and omnivores. After adjusting for age, sex, and total energy intake, vegetarians were found to consume significantly less daily protein compared to omnivores [72.15 g (95% CI: 68.91, 75.39) vs. 78.94 g (95% CI: 75.51, 82.38); *p* = 0.01]. Additionally, protein contributed a significantly greater percentage of the total caloric intake among omnivores than vegetarians (*p* < 0.01). These findings remained significant in the sensitivity analysis, which accounted for normalized dietary variables and excluded outliers (*p* < 0.01).

#### 3.4.2. Carbohydrates

Vegetarians and omnivores had a comparable daily carbohydrate intake, with no significant difference observed in the primary analysis [297.43 g (95% CI: 290.13, 304.73) vs. 287.63 g (95% CI: 279.89, 295.38); *p* = 0.07]. However, the findings from the sensitivity analysis revealed that vegetarians consumed a significantly greater amount of carbohydrates than omnivores [290.06 g (95% CI: 283.25, 297.02) vs. 279.97 g (95% CI: 273.01, 287.11); *p* = 0.05]. In addition, vegetarians obtained a larger proportion of their total caloric intake from carbohydrates compared to omnivores (60.32% ± 5.98 vs. 57.73% ± 6.03; *p* = 0.04). The carbohydrate quality also differed significantly between the groups, with vegetarians consuming carbohydrates with a higher glycemic index and glycemic load (linear regression model: *p* = 0.03 and *p* = 0.01, respectively; sensitivity analysis: *p* = 0.04 and *p* = 0.01, respectively), based on the calculations derived from the individual SHARE FFQ responses.

### 3.5. Dietary Fiber

There were no significant differences between vegetarians and omnivores in their average daily intake of total fiber (*p* = 0.45), soluble fiber (*p* = 0.83), or insoluble fiber (*p* = 0.81). These findings remained non-significant in the sensitivity analysis.

### 3.6. Dietary Fat

There were no significant differences in the total daily mean fat intake between vegetarians and omnivores [67.85 g (95% CI: 65.50, 70.21) vs. 65.92 g (95% CI: 63.42, 68.42); *p* = 0.27]. While 10.4% of the participants exceeded the recommended daily fat intake, no statistically significant differences were observed between the diet groups in their mean intake of saturated fat (*p* = 0.45), monounsaturated fat (*p* = 0.27), polyunsaturated fat (*p* = 0.35), or trans fat (*p* = 0.86). These findings were consistent in the sensitivity analysis. Both vegetarians and omnivores obtained comparable proportions of their total energy intake from fat; however, vegetarians consumed significantly lower amounts of dietary cholesterol than omnivores [100.36 mg (95% CI: 85.61, 115.11) vs. 161.52 mg (95% CI: 145.87, 177.17); *p* < 0.01].

### 3.7. Micronutrient Intake

Significant differences were observed between vegetarians and omnivores in their average daily intake of several micronutrients. In the linear regression analysis, vegetarians had significantly lower intake of niacin [1.91 equivalents (95% CI: 1.62, 2.21) vs. 2.94 equivalents (95% CI: 2.62, 3.25); *p* < 0.01]; vitamin B-12 [2.47 µg (95% CI: 2.15, 2.79) vs. 3.21 µg (95% CI: 2.87, 3.55); *p* < 0.01]; potassium [4091.65 mg (95% CI: 3938.06, 4245.24) vs. 4370.33 mg (95% CI: 4207.39, 4533.26); *p* = 0.02]; and zinc [9.55 mg (95% CI: 9.29, 9.82) vs. 10.18 mg (95% CI: 9.90, 10.47); *p* < 0.01] compared to omnivores. These findings remained statistically significant in the sensitivity analysis, which also revealed that vegetarians consumed significantly more iron than omnivores [16.25 mg (95% CI: 15.62, 16.90) vs. 15.23 mg (95% CI: 14.59, 15.90); *p* = 0.03]. Notably, 74.0% of participants did not meet their daily recommended intake, referred to as adequate intake (AI), for potassium, while daily sodium intake exceeded recom-mended levels in all participants.

### 3.8. Cardiovascular Risk Factors

Vegetarians and omnivores did not significantly differ with respect to their mean BMI (27.7 (±3.9) vs. 28.5 (±3.3) kg/m^2^; *p* = 0.26) or mean WC (100.4 (±10.1) cm vs. 102.8 (±8.3) cm; *p* = 0.21). When comparing vegetarians and omnivores, participants in both groups shared similar adiposity measures (Table 5), and the odds of overweight and obesity were not significant based on the ICS-DOAMS (0.68 (0.16, 2.82)) or WHO (0.53 (0.18, 1.58)) guidelines (Table 6).

## 4. Discussion

In the present study, the usual dietary intake and CVD risk factors were compared between adult SA vegetarians and omnivores living in the Metro Vancouver region. Although the total energy and overall carbohydrate intake did not significantly differ between groups, vegetarians derived a higher proportion of their total energy from carbohydrates. Furthermore, compared to omnivores, vegetarians consumed carbohydrates with a greater glycemic load and glycemic index. As expected, the protein intake was higher among omnivores, whereas the total fat intake, including that of saturated, monounsaturated, polyunsaturated, and trans fats, was comparable between groups.

Our finding that vegetarians obtain a higher percentage of their total energy from carbohydrates aligns with previous studies conducted in the United States (US), the United Kingdom (UK), and India [23,24,35]. For instance, among 4508 SAs in the UK Biobank, Tong et al. reported that vegetarians consumed a higher proportion of their energy from carbohydrates compared to omnivores [24]. Similarly, Shridhar and colleagues found in the India Migration Study that vegetarian participants had a higher daily carbohydrate intake than that of their omnivorous counterparts [35].

While the quantity of carbohydrate intake is important, the type of carbohydrates consumed also plays a significant role in CVD risk. Previous studies have shown that SA diets are often characterized by a high glycemic load and glycemic index [36,37]. In our study, vegetarians consumed carbohydrates with a higher glycemic load and glycemic index than omnivores, a finding consistent with the MASALA study involving 892 SA adults in California and Illinois [23]. In contrast, the Adventist-2 and NHANES studies, which included 91,614 individuals from various ethnic backgrounds, reported that vegetarians consumed fewer high-glycemic-index foods than omnivores [22,38]. These differing results may reflect the increased consumption of processed and convenience foods among SA immigrants, with dietary acculturation potentially contributing to a greater CVD risk in this population [39,40,41].

Both dietary groups in our study had similar intakes of total fat, including saturated and trans fats. Notably, after adjusting for age, sex, and total energy intake, the average saturated fat intake in both groups exceeded the American Diabetes Association (ADA)’s recommended threshold of less than 7% of total energy from saturated fats [42]. Similarly, Tong et al. reported that the mean saturated fat intake among SAs in the UK exceeded 10%, even after adjustment for age [24]. This elevated intake may reflect cultural cooking practices common among SAs, such as the use of ghee, vegetable ghee, and butter in food preparation [43,44].

The fat intake patterns observed in our cohort are in line with that from the Alberta Tomorrow Project (140 SA participants; 77% vegetarian) which reported processed foods high in total fat, trans fat, and refined carbohydrates contributed to approximately 35% of the total energy intake [28]. These findings suggest that a vegetarian diet may involve limiting animal products but can still include the consumption of unhealthy, high-fat foods—potentially increasing diabetes risk. Therefore, the composition of a vegetarian diet may differ significantly between Western and SA cultural contexts.

A high sodium intake has been associated with an elevated risk of hypertension in SA populations [45,46]. As observed in both the MASALA study [23] and the India Migration Study [35], the sodium intake in our cohort exceeded the adequate intake (AI) recommendation of 2300 mg/day [47], regardless of diet group. This could be attributed to cooking practices involving the liberal use of salt and the consumption of processed or refined foods high in sodium [39,40,41]. Given that SAs are more likely to develop hypertension at younger ages and higher rates than White Caucasians [2,3,4,5,6,7,8,9], dietary strategies to reduce sodium intake may be especially critical in this community.

In terms of CVD risk, our study assessed anthropometric indicators and found no significant differences in BMI, WC, or the risk of overweight/obesity between vegetarians and omnivores. These results are consistent with the Diabetes among Indian Americans (DIA) study, which examined 1038 SA adults in the US and reported no increased obesity risk among vegetarians compared to that in omnivores [26]. Using the ICS-DOAMS guidelines adapted for SA populations, only 9.4% (n = 9) of the participants in our study had a normal BMI, while 91% (n = 87) were classified as overweight or obese. According to the standard WHO criteria, 18.8% (n = 18) had a normal BMI, and 81.2% (n = 78) were overweight or obese. Although BMI has limitations in not accounting for fat distribution, age, or sex, it remains a relevant and important marker, particularly for SAs who exhibit worse vascular health and face a higher burden of CVD morbidity and mortality compared to that in non-ethnic populations [48,49]. Thus, the use of culture-specific criteria is essential for the early identification of metabolic syndrome, diabetes, and CVD risk in SA populations.

### Limitations and Future Implications

This study has important limitations that should be acknowledged. First, the use of self-reported dietary assessment tools such as the SHARE Food Frequency Questionnaire (FFQ) introduces potential for social desirability and recall biases. Although the SHARE FFQ is culturally tailored, it was not specifically developed with input from the Sikh Punjabi population and may have omitted relevant food items commonly consumed by this community. As such, future studies might consider using 24 h dietary recall methods, which could offer a more culturally sensitive and accurate reflection of dietary intake while reducing recall bias. Additionally, due to the limited sample size, the vegetarian group included all vegetarian diets (e.g., vegan, lacto-vegetarian), which restricted our ability to explore potential differences in the dietary intake among specific vegetarian subgroups. Second, the cross-sectional design of this study limits the ability to draw causal inferences between dietary intake and CVD risk factors. Longitudinal observational studies are warranted to understand the long-term health impacts of dietary intake in this population better. Third, the sample size was relatively small and predominantly consisted of older immigrant SA adults who self-identified as Sikh Punjabi, with a higher proportion of female participants in the vegetarian group compared to that among omnivores. These demographic characteristics may limit the generalizability of our findings to the broader Canadian SA population. Fourth, this study focused solely on anthropometric measures and did not include additional CVD risk indicators such as blood pressure, lipid profiles, or markers of glycemic control. The absence of these measures restricts the comprehensiveness of our cardiovascular risk assessment. Finally, while the broader Prevention Matters study collected data on physical activity and psychosocial variables, these factors were not analyzed in the current study. Future research should integrate these variables to enable a more complete understanding of energy balance and dietary behavior. Including a broader range of CVD risk markers beyond anthropometry would also allow for a more holistic evaluation of cardiovascular and metabolic health measures.

## 5. Conclusions

Diet is an important modifiable lifestyle factor that can reduce CVD risk and diabetes. While adherence to a vegetarian diet has proven to be beneficial for metabolic health in various ethnic groups, it appears that these health benefits do not uniformly apply to the SA community. In other words, “not all vegetarian diets are created equal” and thus cannot be assumed to be universally healthy. Most importantly, regardless of preference for a vegetarian or omnivorous diet, this SA sample reported carbohydrate, fat, and sodium intake levels that place the community, on the whole, at greater CVD risk. That over 90% of all of the study participants were classified as obese or overweight only confirms this observation. Considering the early and heightened CVD risk experienced by SAs, dietary interventions that will have the greatest impact need to target both vegetarians and omnivores.

## Figures and Tables

**Table 1:** Sociodemographic characteristics, BMIs, and WCs of vegetarian and non-vegetarian study participants.

	Omnivore (n = 46)	Vegetarian (n = 50)	*p*-Value
Age (Years)	65.6 ± 10.5 [Median (IQR) 65.0 (60.0, 73.0)]	64.9 ± 9.0 [Median (IQR) 63.5 (58.0, 71.0)]	0.72
Sex (%)			0.06
Female	34.8	54.0	
Male	65.2	46.0	
Marital Status (%)			0.86
Married	88.9	89.8	
Never Married	0.0	2.0	
Widowed	11.1	8.2	
First Language (%)			0.07
English	71.1	51.0	
Hindi	0.0	2.0	
Punjabi	28.9	46.9	
Religion (%)			0.64
Hindu	11.1	14.3	
Sikh	88.9	85.7	
Years Lived in Canada ^a^	27.8 ± 13.2	22.5 ± 14.5	0.07
Education Level (%)			0.26
<High School	35.6	46.9	
High School	37.8	22.4	
>High School	26.7	30.6	
Annual Household Income (%)			0.92
<CAD 20,000	28.6	27.9	
CAD 20,000–CAD 49,999	42.9	39.5	
>CAD 50,000	28.6	32.6	
Employed (%)	28.9	20.4	0.34
Smoking Status (%)			0.48
<1 Year Ago	2.2	100.0	
Never	97.8	0.0	
BMI (kg/m^2^)	28.5 ± 3.3 [Median (IQR) 28.8 (26.1, 30.8)]	27.7 ± 3.9 [Median (IQR) 27.0 (25.8, 29.8)]	0.26
WC (cm)	102.8 ± 8.3 [Median (IQR) 101.6 (97.8, 108.0)]	100.4 ± 10.1 [Median (IQR) 99.4 (94.0, 106.7)]	0.21

^a^ Data for years lived in Canada were available for 43 omnivore participants and 48 vegetarian participants.

**Table 2:** Daily adjusted mean nutrient intakes between vegetarian and non-vegetarian study participants.

	Omnivore (n = 46)	Vegetarian (n = 50)	*p*-Value
Energy Intake (Calories)	2068.98 (1915.35, 2222.60)	1937.84 (1793.53, 2082.15)	0.22
Protein (g)	78.94 (75.51, 82.38)	72.15 (68.91, 75.39)	0.01
Carbohydrates (g)	287.63 (279.89, 295.38)	297.43 (290.13, 304.73)	0.07
Glycemic Load	110.41 (104.55, 116.26)	121.07 (115.55, 126.60)	0.01
Glycemic Index	42.29 (40.88, 43.70)	44.47 (43.14, 45.80)	0.03
Total Fiber (g)	26.08 (24.48, 27.67)	26.93 (25.42, 28.43)	0.45
Soluble Fiber (g)	10.86 (10.23, 11.49)	10.76 (10.17, 11.36)	0.83
Insoluble Fiber (g)	11.08 (10.20, 11.95)	10.93 (10.11, 11.76)	0.81
Total Fat (g)	65.92 (63.42, 68.42)	67.85 (65.50, 70.21)	0.27
Saturated Fat (g)	20.88 (19.30, 22.45)	21.71 (20.22, 23.19)	0.45
Monounsaturated Fat (g)	24.74 (23.55, 25.93)	25.66 (24.53, 26.79)	0.27
Polyunsaturated Fat (g)	13.54 (12.81, 14.26)	14.01 (13.33, 14.70)	0.35
Trans Fat (g)	0.37 (0.23, 0.51)	0.35 (0.22, 0.49)	0.86
Cholesterol (mg)	161.52 (145.87, 177.17)	100.36 (85.61, 115.11)	<0.01
Vitamin A (IU)	18,945.00 (16,422.00, 21,468.00)	16,269.00 (13,890.00, 18,647.00)	0.13
Niacin (NE)	2.94 (2.62, 3.25)	1.91 (1.62, 2.21)	<0.01
Vitamin B-6 (mg)	2.29 (2.17, 2.42)	2.22 (2.11, 2.34)	0.43
Vitamin B-12 (µg)	3.21 (2.87, 3.55)	2.47 (2.15, 2.79)	<0.01
Vitamin C (mg)	259.31 (230.59, 288.02)	222.71 (195.64, 249.78)	0.07
Vitamin D (IU)	113.23 (89.07, 137.39)	102.36 (79.59, 125.13)	0.52
Vitamin E (IU)	2.12 (1.79, 2.46)	2.00 (1.69, 2.32)	0.61
Folate (µg)	479.57 (450.37, 508.77)	459.15 (431.62, 486.68)	0.32
Calcium (mg)	1161.25 (1071.51, 1250.99)	1071.42 (986.83, 1156.02)	0.16
Chromium (µg)	14.94 (12.76, 17.11)	14.84 (12.79, 16.89)	0.95
Iron (mg)	16.27 (15.16, 17.38)	16.93 (15.89, 17.98)	0.39
Potassium (mg)	4370.33 (4207.39, 4533.26)	4091.65 (3938.06, 4245.24)	0.02
Selenium (mg)	10.83 (7.08, 14.59)	7.94 (4.39, 11.48)	0.27
Sodium (mg)	3367.74 (3146.52, 3588.96)	3163.51 (2954.97, 3372.04)	0.19
Zinc (mg)	10.18 (9.90, 10.47)	9.55 (9.29, 9.82)	<0.01
Omega-3 Fatty Acid (g)	0.15 (0.11, 0.20)	0.12 (0.08, 0.16)	0.27
Omega-6 Fatty Acid (g)	0.51 (0.40, 0.63)	0.58 (0.47, 0.69)	0.43

Statistical model adjusted for age, sex, and energy intake.

**Table 3:** Daily adjusted mean nutrient intakes between vegetarian and non-vegetarian study participants with log transformation.

	Omnivore (n = 46)	Vegetarian (n = 50)	*p*-Value
Energy Intake (Calories)	1995.71 (1851.62, 2151.01)	1873.84 (1746.47, 2010.49)	0.23
Protein (g)	74.64 (71.53, 77.88)	67.56 (64.93, 70.30)	<0.01
Carbohydrates (g)	279.97 (273.01, 287.11)	290.06 (283.25, 297.02)	<0.05
Glycemic Load	107.36 (102.24, 112.73)	117.11 (111.85, 122.63)	0.01
Glycemic Index	42.04 (40.66, 43.46)	44.15 (42.79, 45.56)	0.04
Total Fiber (g)	24.50 (23.22, 25.85)	25.84 (24.58, 27.17)	0.16
Soluble Fiber (g)	10.32 (9.77, 10.89)	10.44 (9.92, 10.98)	0.76
Insoluble Fiber (g)	10.31 (9.62, 11.05)	10.38 (9.72, 11.07)	0.89
Total Fat (g)	62.06 (59.76, 64.45)	63.30 (61.09, 65.60)	0.45
Saturated Fat (g)	19.26 (17.92, 20.70)	19.62 (18.33, 20.99)	0.72
Monounsaturated Fat (g)	23.20 (22.11, 24.33)	23.63 (22.59, 24.72)	0.58
Polyunsaturated Fat (g)	12.71 (12.10, 13.35)	13.18 (12.59, 13.80)	0.29
Trans Fat (g)	0.17 (0.12, 0.24)	0.18 (0.13, 0.25)	0.85
Cholesterol (mg)	140.14 (125.77, 156.15)	83.80 (75.68, 92.80)	<0.01
Vitamin A (IU)	16,108.00 (14,014.91, 18,513.68)	14,165.82 (12,423.70, 16,152.23)	0.19
Niacin (NE)	2.46 (2.23, 2.71)	1.66 (1.52, 1.81)	<0.01
Vitamin B-6 (mg)	2.16 (2.07, 2.26)	2.10 (2.02, 2.19)	0.37
Vitamin B-12 (µg)	2.83 (2.54, 3.16)	2.11 (1.90, 2.34)	<0.01
Vitamin C (mg)	228.17 (204.23, 254.92)	203.70 (183.48, 226.14)	0.15
Vitamin D (IU)	85.83 (67.29, 109.48)	72.86 (57.92, 91.65)	0.34
Vitamin E (IU)	1.75 (1.56, 1.95)	1.81 (1.63, 2.00)	0.63
Folate (µg)	456.84 (434.17, 480.69)	445.50 (424.43, 467.61)	0.48
Calcium (mg)	1099.30 (1020.36, 1184.35)	1002.69 (934.66, 1075.66)	0.08
Chromium (µg)	12.79 (11.14, 14.69)	12.80 (11.23, 14.58)	0.99
Iron (mg)	15.23 (14.59, 15.90)	16.25 (15.62, 16.90)	0.03
Potassium (mg)	4183.91 (4029.09, 4344.68)	3918.34 (3782.77, 4058.76)	0.01
Selenium (mg)	7.76 (6.92, 8.71)	6.59 (5.92, 7.34)	0.04
Sodium (mg)	3098.47 (2915.24, 3293.20)	2978.34 (2813.54, 3152.80)	0.35
Zinc (mg)	9.87 (9.58, 10.16)	9.19 (8.94, 9.45)	<0.01
Omega-3 Fatty Acid (g)	0.11 (0.10, 0.13)	0.09 (0.08. 0.11)	0.07
Omega-6 Fatty Acid (g)	0.44 (0.37, 0.51)	0.45 (0.39, 0.53)	0.74

Statistical model adjusted for age, sex, and energy intake (log transformation excluding outliers).

**Table 4:** Estimated proportions of daily calories from protein, carbohydrates, and fat between vegetarian and non-vegetarian study participants.

	Omnivores (n = 46)	Vegetarians (n = 50)	*p*-Value
Protein (%)	15.62 (±2.21)	14.13 (±2.07)	<0.01
Carbohydrates (%)	57.73 (±6.03)	60.32 (±5.98)	0.04
Fat (%)	28.93 (±4.13)	29.40 (±4.83)	0.61

**Table 5:** BMI categorization of study participants based on ICS-DOAMS and WHO guidelines.

	BMI Category	Omnivore	Vegetarian	*p*-Value
ICS-DOAMS	Normal (%)	4 (8.7)	5 (10.0)	0.74
	Overweight (%)	3 (6.5)	6 (12.0)	
	Obese (%)	39 (84.8)	39 (78.0)	
WHO	Normal (%)	7 (15.2)	11 (22.0)	0.34
	Overweight (%)	23 (50.0)	28 (56.0)	
	Obese (%)	16 (34.8)	11 (22.0)	

**Table 6:** Odds of overweight and obesity in vegetarians versus omnivores based on ICS-DOAMS and WHO guidelines.

	Odds Ratio for Overweight/Obesity (Vegetarian to Omnivore)	*p*-Value *
ICS-DOAMS	0.68 (0.16, 2.82)	0.59
WHO	0.53 (0.18, 1.58)	0.25

* Statistical model adjusted for age and sex.

## Data Availability

The original contributions presented in this study are included in the article. Further inquiries can be directed to the corresponding author.
